# Characterization of Porous CuO Films for H_2_S Gas Sensors

**DOI:** 10.3390/ma15207270

**Published:** 2022-10-18

**Authors:** Dawoon Jung, Sehoon Hwang, Hyun-Jong Kim, Jae-Hee Han, Ho-Nyun Lee

**Affiliations:** 1 Heat & Surface Technology R&D Department, Korea Institute of Industrial Technology (KITECH), Incheon 21999, Korea; 2 Department of Materials Science and Engineering, Gacheon University, Seongnam-si 13120, Korea

**Keywords:** porous nanoparticle, copper oxide, thermal evaporation, porosity, gas sensor, H_2_S detection

## Abstract

Using a thermal evaporator, various porous Cu films were deposited according to the deposition pressure. CuO films were formed by post heat treatment in the air. Changes in morphological and structural characteristics of films were analyzed using field-emission scanning electron microscopy (FE-SEM) and X-ray diffraction (XRD). Relative density and porosity were quantitatively calculated. CuO films with various pores ranging from 39.4 to 95.2% were successfully manufactured and were applied as gas sensors for H_2_S detection on interdigitated electrode (IDE) substrate. Resistance change was monitored at 325 °C and an increase in porosity of the film improved the sensor performance. The CuO-10 gas sensor with a high porosity of 95.2% showed a relatively high response (2.7) and a fast recovery time (514 s) for H_2_S 1.5 ppm. It is confirmed that the porosity of the CuO detection layer had a significant effect on response and recovery time.

## 1. Introduction

Recently, the creation of a safe and healthy environment has been attracting increasing attention. The monitoring of toxic and harmful substances or air quality is considered to be increasingly important [1,2]. For this type of monitoring, metal oxide nanomaterials are being applied due to their low production costs and the possibility of different characterizations according to different sizes and morphologies such as nanotubes and nanowires [3,4,5]. Nano-porous structures are particularly suitable for high-response sensing due to their high surface-to-volume ratios [6,7,8,9].

CuO is a promising material for hydrogen sulfide (H_2_S) gas detection. H_2_S is a common toxic gas that is released as a result of various industrial activities and food spoilage. Even a very small amount of H_2_S is very harmful to the human body, causing high explosiveness, respiratory and eye irritation, sore throat, nervous system paralysis, and loss of consciousness [10,11]. Therefore, research on H_2_S sensors with high response—even at concentrations as low as ppb—is currently in high demand. According to results of research examining CuO as a H_2_S gas sensing material [8,12,13,14], CuO can react with H_2_S and recover quickly at high temperatures. However, at low temperatures, re-oxidation of CuO is difficult. Thus, it encounters limitations such as slow recovery and irreversible behavior. It is therefore important to select the appropriate temperature for both response and recovery.

Meanwhile, in order to understand the relationship between response and recovery time according to porosity of metal oxides, studies have been conducted to investigate effects of porosity and various shapes of nanoparticles [15,16,17,18]. Although previous studies have reported sensor characteristics according to various porosity, the porosity change range was narrow [17,18]. Therefore, it is necessary to investigate the porosity effect over a wide range.

In this study, we prepared Cu and CuO films with a wide range of porosity using a thermal evaporator to investigate correlation between porosity and sensor performance. Using heat treatment as an oxidation method, we successfully fabricated a porous CuO structure and applied it as a resistance change gas sensor for H_2_S.

## 2. Materials and Methods

### 2.1. Preparation of Porous Cu Films

Porous Cu films were fabricated through evaporation of a Cu source (0.6 g, 99.999%, LTS Research Lab.) using a chamber-type thermal evaporation system (ULTEC). The Cu source was placed at the bottom center of the chamber in the W boat. Prior to the deposition process, the Si substrate was cleaned with acetone, isopropyl alcohol, and deionized water for 10 min each and then dried by N_2_ blowing. The cleaned substrate placed on top was rotated at 8 rpm with cooling at 19 °C during deposition. The source and substrate were fixed at a distance of 12 cm. After the evacuation of the vacuum chamber to within 5 × 10^−6^ Torr, the specified deposition pressure was maintained by a throttle valve with pure Ar gas (99.9999%) at a 100 sccm flow. These porous Cu films were deposited under different pressures (0.05, 0.2, 0.5, and 1.0 Torr) and then heat-treated at temperatures ranging from 400 to 700 °C in 0.5 slm dry air for 1 h in a tube furnace for oxidation of porous Cu films. These porous Cu films before heat treatment were denoted as Cu-005, Cu-02, Cu-05, and Cu-10. Corresponding porous CuO films after heat treatment were, respectively, denoted as CuO-005, CuO-02, CuO-05, and CuO-10.

### 2.2. Characterization of Porous Films

To observe morphologies and thicknesses of porous Cu and CuO films, field-emission scanning electron microscopy (FE-SEM, FEI Quanta 200F) analysis was conducted. Thickness was determined using a parallel line to the substrate to specify the average position of film heights in the SEM image, and the distance between the line and the substrate was then measured. Densities ($\rho_{film})$ of porous Cu and CuO were determined by measuring weight change before and after deposition/heat treatment and dividing by volume. The volume was calculated from the area of the porous film and the thickness derived from the SEM. The porosity was obtained with Equation (1), where ($\rho_{film}$/$\rho_{bulk}$) meant the ratio of the film density to the bulk density and defined as the relative density [19]. The density of bulk-type Cu and CuO are 8.96 [20] and 6.31 g/cm^3^ [21], respectively. The crystal structure was evaluated through X-ray diffraction (XRD, Panalytical X’Pert PRO MPD) using Cu K_α_ radiation. The size of nanoparticles was measured by randomly selecting 20 particles in the FE-SEM cross-sectional image. The length of each particle was then measured and averaged through a measurement program.

(1) $$\mathrm{Porosity}=\left(1-\left(\frac{\rho_{film}}{\rho_{bulk}}\right)\right)\times 100\;(\%)$$

### 2.3. Fabrication and Testing of Gas Sensors

To prepare a gas sensor for H_2_S detection, a 30 nm Cr/150 nm Pt electrode was deposited in an interdigitated electrode (IDE) pattern on a 2 × 2 mm^2^ Si wafer (p-type) coated with 300 nm-thick SiO_2_ and 200 nm-thick Si_3_N_4_. Because the delamination between the Pt/Cr electrode and SiO_2_ layer occurred during the lift-off process owing to the poor adhesion, the Si_3_N_4_ coating layer was added to solve this problem. Cu films were deposited using a shadow mask, followed by heat treatment. Three gases of N_2_ (6N), O_2_ (5N), and N_2_ balanced H_2_S of 100 ppm were used for gas test. A specific concentration was prepared by mixing with H_2_S gas (N_2_ balance, 100 ppm) and dry air (79% N_2_ gas, 21% O_2_ gas) and total gas flow was fixed at 200 sccm. Gas was injected into the test chamber and vented out. The existing gas in the chamber could be replaced with the injected gas in 1 min theoretically, because the volume of the chamber was less than 100 cc. Thus, the relative humidity of the chamber was 0%, and change in resistance was monitored using a Keithley 2600 A instrument. In this paper, the response of the gas sensor was defined as S = R_g_/R_a_, where R_g_ was the maximum resistance value in H_2_S gas reaction and R_a_ was the resistance value stabilized in dry air. Response time and recovery time were analyzed by observing the time required to reach 90% of total resistance change.

## 3. Results and Discussion

### 3.1. Fabrication of Porous Cu and CuO Films

**Figure 1:**
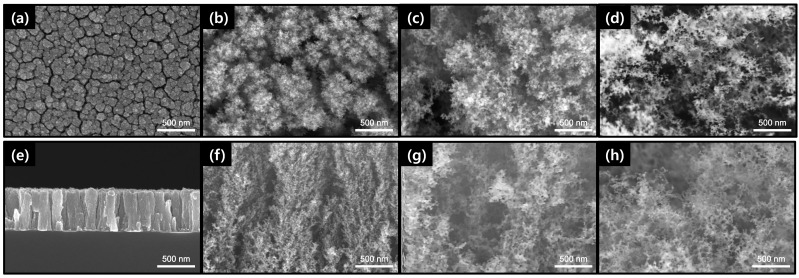
FE-SEM (**a**–**d**) surface and (**e**–**h**) cross-section images of porous Cu films with various process pressure: (**a**,**e**) 0.05 Torr, (**b**,**f**) 0.2 Torr, (**c**,**g**) 0.5 Torr, and (**d**,**h**) 1.0 Torr.

Figure 1 shows FE-SEM surface and cross-sectional images of porous Cu films deposited at various deposition pressures. The Cu-005 film had a relatively dense and columnar structure. A number of gaps could be observed between columnar structures. Except for Cu-005, the rest of the porous Cu films showed clustered nanoparticles and resembled short fluffs. As the deposition pressure increased, there was an increase in the spacing as if the fluff was torn down and large voids were also observed. This result could be attributed to the fact that the selected process pressures were higher than typical deposition pressures (<1 × 10^−5^ Torr) in the evaporation process. The high pressure led to many collisions of evaporated Cu particles with Cu particles and Ar gas before they were deposited on the substrate. Therefore, they reached the substrate with a low energy, at which point they did not have sufficient surface diffusion [17,19].

**Figure 2:**
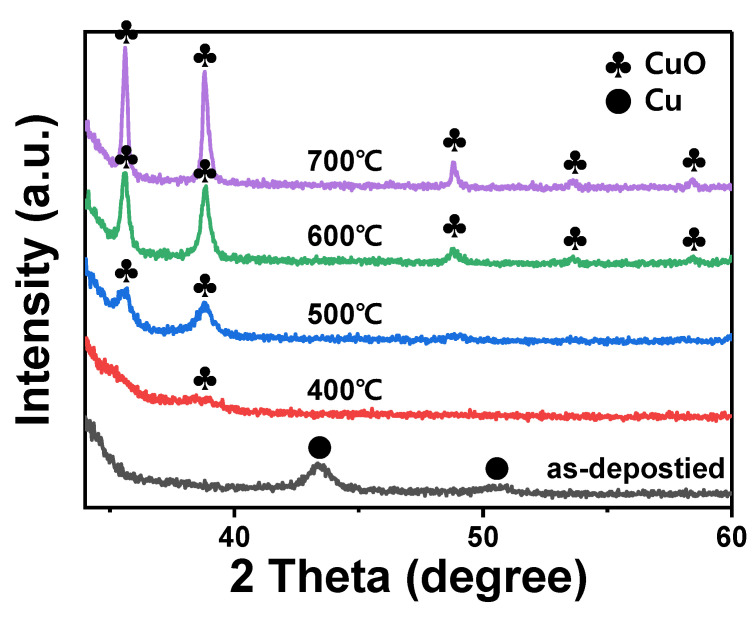
XRD patterns of porous Cu-05 films as-deposited and after heat treatment at different temperatures.

Heat treatment was conducted to oxidize the porous Cu thin film. To minimize particle coarsening due to Cu diffusion, the temperature was raised at a rate of 5 °C/min to the target temperature. To investigate the crystal structure, XRD analysis of the Cu-05 film was conducted before and after heat treatment at temperatures ranging from 400 to 700 °C (Figure 2). The as-deposited Cu film showed peaks with 2 theta values of 43.4 and 50.7° corresponding to (111) and (200) phases of Cu, respectively [22]. After heat treatment, the (111) and (200) Cu peaks disappeared, while other peaks appeared. As the temperature increased, it could be seen that two peaks with 2 theta values of 35.6 and 38.8° corresponding to (−111) and (111) planes of CuO, respectively, were sharpened. Moreover, other peaks at 48.8, 53.6, and 58.4° corresponding to (−202), (020), and (202) planes of CuO, respectively, showed low intensities [23,24]. Based on these results, CuO-05 film was determined to have a monoclinic CuO structure.

**Figure 3:**
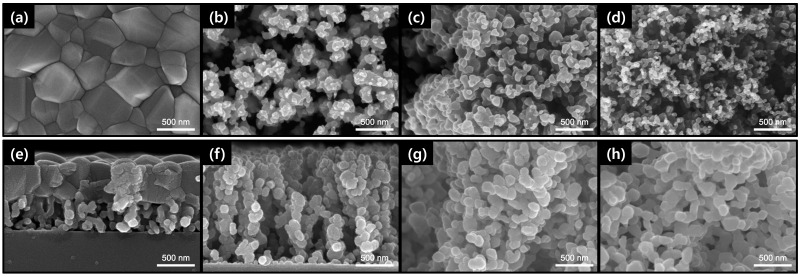
FE-SEM (**a**–**d**) surface and (**e**–**h**) cross-sectional images of porous CuO films heat treated at 600 °C with various process pressure: (**a**,**e**) 0.05 Torr, (**b**,**f**) 0.2 Torr, (**c**,**g**) 0.5 Torr, and (**d**,**h**) 1.0 Torr.

Figure 3 shows FE-SEM surface and cross-sectional images of porous CuO films after heat treatment at a temperature of 600 °C. The surface morphology of the CuO-005 film seemed to be densely packed with clumps of several hundred nanometers. On the other hand, the cross-section was composed of an upper layer formed in a lump and a lower layer having a high porosity due to out-diffusion of Cu as shown in Figure 3e. Moreover, the CuO-005 film of 1.0 µm was thicker than the Cu-005 film of 0.6 µm due to added oxygen atoms. Except for CuO-005, CuO particles in the films had grown in size and agglomerated together during heat treatment. Each CuO film with thickness of 1.6, 4.4, and 7.7 µm became thinner than each Cu film with thickness of 2.2, 6.6, and 26.4 µm, respectively. This was attributed to the presence of sufficient porosity to accommodate volume expansion and coarsening of particles during the reaction between Cu and O atoms. While CuO-005 consists of a two-layer structure, CuO-02, CuO-05, and CuO-10 appear to have groups of nanoparticles similar to grape clusters. Surface images showed that the sizes of nanoparticles decreased as process pressure increased. However, cross-sectional images showed that nanoparticle sizes of CuO-02, CuO-05, and CuO-10 were almost the same at about 80 to 100 nm. Moreover, the crystallite size by Scherrer’s equation was also almost similar from 25 to 30 nm except the CuO-005 film.

**Figure 4:**
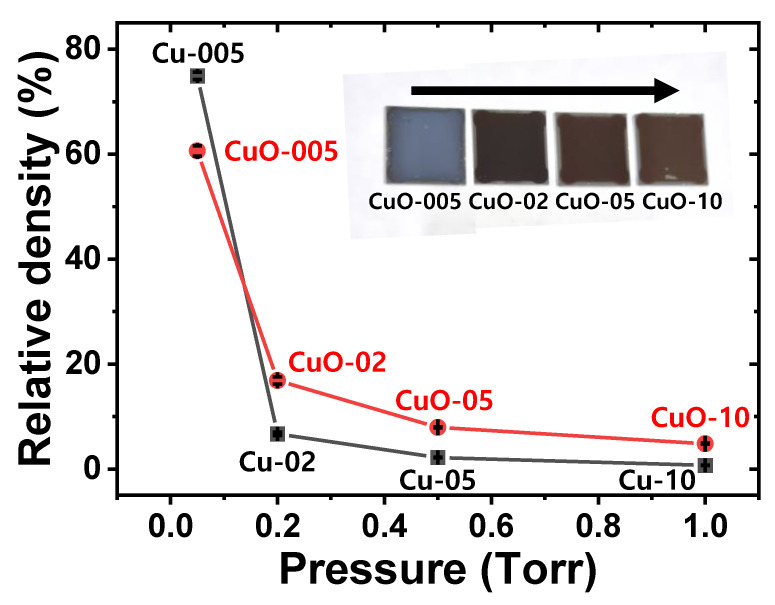
Relative density graph according to deposition pressure of Cu and CuO films heat treated at 600 °C. Inset shows optical photographs of CuO samples.

The relative density was calculated based on the mass and thickness obtained from SEM analysis before and after heat treatment at 600 °C. As shown in Figure 4, porous Cu and CuO films had relative density values ranging from 0.7 to 74.9% and from 4.8 to 60.6%, respectively. With increasing pressure at 0.05, 0.2, 0.5, and 1.0 Torr, porosity values of Cu films were 25.1, 93.3, 97.8, and 99.3%, respectively, and porosity values of CuO films were 39.4, 83.1, 92.1 and 95.2%, respectively. As shown in Figure 1 and Figure 3, relative densities of CuO-02, CuO-05, and CuO-10 films were higher than those of Cu-02, Cu-05, and Cu-10 films due to thickness reduction and volume expansion during oxidation, whereas relative density of CuO-005 film was lower than that of relatively dense Cu-005. As a result, Cu and CuO porosities were clearly controlled in a wide range by adjusting the process pressure, which could lead to a greater change in porosity, particularly in the lower pressure range (~0.2 Torr). Moreover, as the pressure increased, CuO films changed from bluish gray to brown, which could be attributed to scattering from the porous structure.

**Figure 5:**
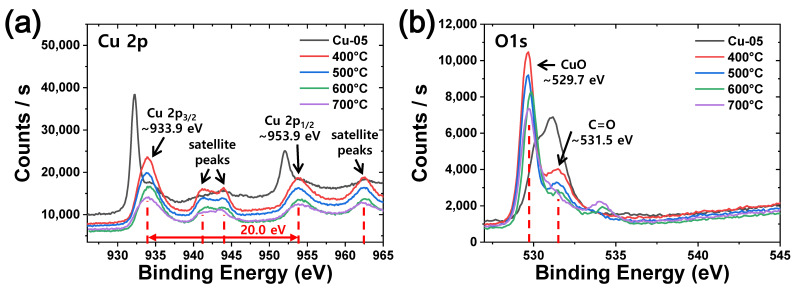
XPS spectra of Cu-05 film and CuO-05 films heat treated at various temperatures: Cu 2p (**a**) and O 1s (**b**).

In addition, X-ray photoelectron spectroscopy (XPS) analysis of CuO-05 films heat-treated at various temperatures was performed (Figure 5). The results of Cu-05 film are also denoted for comparison. As a result, Cu 2p peaks separated by Cu 2p^3/2^ and Cu 2p^1/2^ were observed at 933.9 and 953.9 eV, respectively. The distance between major peak positions of Cu 2p was 20.0 eV. Additionally, wider satellite peaks appeared at higher binding energies than main peaks. It was found that the satellite peak at 962.6 eV had an interval of 8.7 eV from the main peak (Cu 2p^1/2^). All these results suggest that all heat-treated films are in the CuO state, and this result is consistent with the previous result as shown in Figure 2. In addition, in the XPS spectrum of O 1s, a peak clearly indicating CuO was observed at 529.7 eV, confirming C=O at 531.5 eV.

### 3.2. Gas Sensing Tests for H_2_S

**Figure 6:**
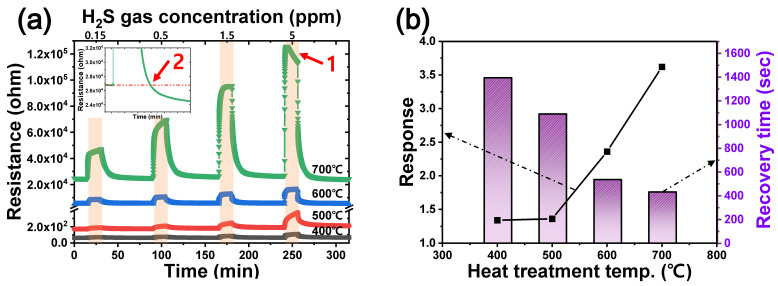
Sensor performance as a function of heat treatment temperature of CuO-05 sensor: (**a**) behavior for various H_2_S gas concentrations at 325 °C and (**b**) response and recovery time for representative gas concentrations (1.5 ppm H_2_S). The inset of (**a**) is a magnified image of the plot to illustrate recovery of CuO-05 heat treated at 700 °C.

Porous CuO films were prepared as a gas detection layer on an IDE substrate. Resistance change was then monitored at 325 °C using our gas sensing system to detect various concentrations of H_2_S gas (Figure S1 and Table S1). First, porous CuO-05 gas sensors were prepared by heat treatment at temperatures ranging from 400 to 700 °C and tested to evaluate gas sensing performance according to heat treatment temperature (Figure 6). The sensor provides a stable resistance baseline in a dry air environment prior to H_2_S detection measurements. H_2_S gas injection increased the resistance, and upon completion of this injection, the resistance decreased. This is a typical sensor behavior of CuO, a p-type semiconductor, based on chemical reactions 2 and 3 as shown below [13,25,26]:

In H_2_S (response): CuO + H_2_S → CuS + H_2_O (2)

In air (recovery): 2CuS + 3O_2_ → 2CuO + 2SO_2_ (3)

Porous CuO films that were heat-treated at 400 and 500 °C showed significantly low response and long recovery time, with respective values of about 1.4 and over 1000 s for 1.5 ppm (Table S2). Porous CuO films oxidized at higher temperature showed improved performance as depicted in Figure 6. These results indicate that the heat-treatment temperature of CuO films is an important factor for determining the gas sensing characteristics. However, in the case of CuO-05 heat-treated at 700 °C, the resistance increased and then decreased, even with continuous injection of high concentrations (>5 ppm) of gas (indicated as ‘1’ in Figure 6a), after which it fell further than the initial R_a_ value (indicated as ‘2’ in the inset of Figure 6a). Kim et al. [27] have explained these abnormal behaviors as a result of gas-induced inversion. Peng et al. [26] have reported that the presence of the metallic CuS layer on the surface of the detection layer could decrease the resistance. In addition, in the case of CuO-10, which had similar structure and particle size with CuO-05 according to the heat treatment temperature (Figures S2 and S3), the sensor heat treated at 700 °C did not return to the initial resistance (Figure S4). As a result, it was determined that the porous CuO gas sensor heat treated at 600 °C had the best performance considering the behavior of resistance change and characteristics of gas sensors.

Further, the operation temperature was optimized over a range of temperatures from 250 to 400 °C (Figure S5). At operating temperatures below 300 °C, the CuO-05 sensor exhibited the above-mentioned abnormal behavior. Lower response and slower recovery were observed with increasing temperature. As a result, the optimal operating temperature was determined to be 325 °C.

**Figure 7:**
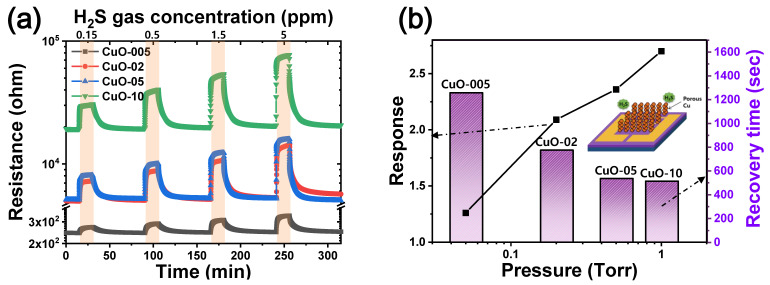
Sensor performance of CuO sensors with different deposition pressures: (**a**) behavior for various H_2_S gas concentrations at 325 °C and (**b**) response and recovery time for representative gas concentrations (1.5 ppm H_2_S).

To investigate the relationship between porosity and sensor performance, Cu films deposited at different deposition pressures were heat treated at 600 °C before being applied as sensors. As shown in Figure 7a, the more porous CuO gas sensor had higher initial resistance (R_a_) values. In particular, the resistance of the CuO-02 sensor did not fully return to its R_a_ within the next gas injection time at high gas concentrations. Gas detection performances at H_2_S concentration of 1.5 ppm are depicted in Figure 7b. As the process pressure increased, the response and recovery time of CuO film increased and decreased, respectively. Responses of CuO-005, CuO-02, CuO-05, and CuO-10 gas sensors were 1.3, 2.1, 2.4, and 2.7, respectively. Meanwhile, the recovery time of CuO-005, CuO-02, CuO-05, and CuO-10 gas sensors were 1257, 775, 536, and 514 s, respectively (Table S3). Among them, the CuO-10 sensor had the best response and recovery time. Furthermore, the response of CuO-10 at H_2_S concentration of 0.5 ppm was 2.1, which is similar to that of a nanowire CuO sensor with a high specific surface area operated at the same temperature as ours [14,28]. Moreover, nanowires were in mechanical contact, whereas our sensor was judged to be much more reliable, as particles were connected by the growth of grains [17].

**Figure 8:**
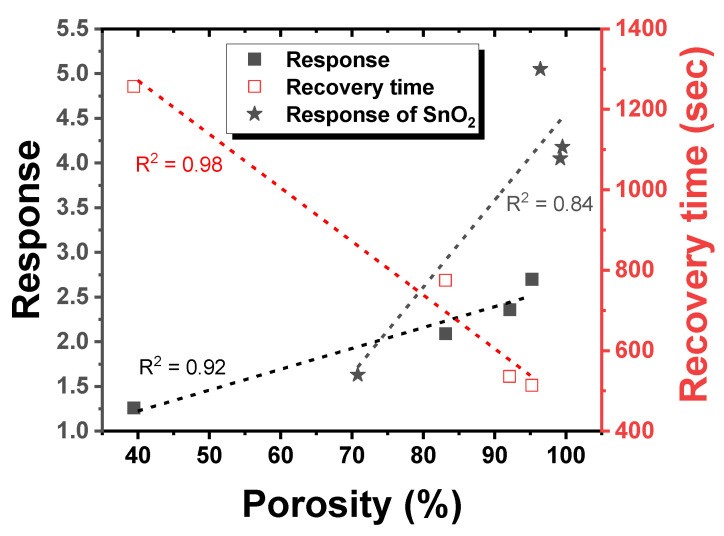
Response and recovery time graphs of CuO sensors heat treated at 600 °C with different porosities using a linear fit curve (H_2_S 1.5 ppm). Comparisons with porous SnO_2_ sensors are included (CO 5 ppm, Han et al., 2019 [17]).

Figure 8 shows characteristics of each sample according to porosity. With increasing porosity from 39.4 to 95.2%, the response increased from 1.3 to 2.7, respectively, and the recovery time decreased from 1257 to 514 s, respectively. They have linear relationships and R^2^ were 0.92 and 0.98 for response and recovery time, respectively, along with the porosity. The high porosity could allow reaction gas to diffuse more easily in and out of the film. Thus, the increased reaction volume of the film enhanced the response of the sensor and the high diffusivity of the reaction gas decreased the recovery time. In our previous research [17], the increase in porosity for the SnO_2_ film from 70.8 to 99.2% can enhance sensor properties, consistent with results of this study. Boarino et al. [18] have also reported that porosity and specific surface area are important variables determining gas sensing characteristics. We used thermal evaporation to deposit films with various porosities and confirmed that films had similar particle sizes except for CuO-005. Based on these results, we could investigate porosity effects on gas sensing characteristics while excluding other factors. Therefore, these results serve as evidence that controlling the porosity of the detection layer is an important factor that can improve the response.

On the other hand, at a gas concentration of more than 5 ppm and an operating temperature of 250 °C or less, the resistance tended to decrease more than the initial resistance value during the reaction and recovery process. While the CuS formation and the inversion could be considered for these reasons as described previously [26,27], a detailed study on these is needed later.

## 4. Conclusions

To investigate the effect of porosity of the detection layer on gas sensing properties, Cu films with different porosities were successfully prepared using thermal evaporation. Heat treatment of porous Cu films was conducted to prepare porous CuO films. Porosities of Cu and CuO were largely controlled by the process pressure from 0.05 to 1.0 Torr, and a wide range of porosity (74.2 and 55.8%, respectively) was realized. Based on results of the H_2_S gas sensing test, heat treatment and operation temperatures were optimized at 600 and 325 °C, respectively. As the deposition pressure of CuO film increased, gas sensing characteristics were enhanced. Among them, CuO-10 heat-treated at 600 °C showed the best performance such as a response of 2.7 and a recovery time of 514 s at H_2_S concentration of 1.5 ppm. In our study, with the exception of CuO-005, CuO particle sizes were similar, so only the porosity effect on sensing performance could be investigated. As a result, the porosity of the detection layer of CuO played an important role. It showed a linear relationship with response and recovery time. Future research into CuS formation and inversion in our porous CuO film should be conducted while obtaining deeper insight into H_2_S gas sensor applications. Studies on effects of humidity and selectivity need to be conducted in the future.

## Data Availability

Not applicable.

## Supplementary Materials

The following supporting information can be downloaded at: https://www.mdpi.com/article/10.3390/ma15207270/s1. Figure S1: Schematic of gas sensing system; Table S1: Composition of gas mixture for various H_2_S concentration; Table S2: Characteristics of CuO-05 sensors heat treated at different temperatures; Table S3: Characteristics of CuO sensors deposited at various deposition pressures and then heat treated at 600 °C; Figure S2: FE-SEM (a–d) surface and (e–h) cross-sectional images of CuO-05 films heat treated at 400, 500, 600, and 700 °C, respectively; Figure S3: FE-SEM (a–d) surface and (e–h) cross-sectional images of CuO-10 films heat treated at 400, 500, 600, and 700 °C, respectively; Figure S4: Resistance change graph for various H_2_S concentrations of CuO-10 heat treated at 700 °C (operating temperature: 325 °C); Figure S5: Sensor performance as a function of operating temperature of CuO-05 heat treated at 600 °C: (a) behavior, (b) response, and (c) recovery time for various H_2_S gas concentrations.
