# Supplementary material for: Characterization of Porous CuO Films for H2S Gas Sensors
Source: Materials (Basel). 2022 Oct 18;15(20):7270. doi: 10.3390/ma15207270 (PMC9610780; doi:10.3390/ma15207270)
Supplement: Supplementary file 1 [file materials-15-07270-s001.zip › materials-1897368-supplementary.pdf]

Supporting Information—Figures

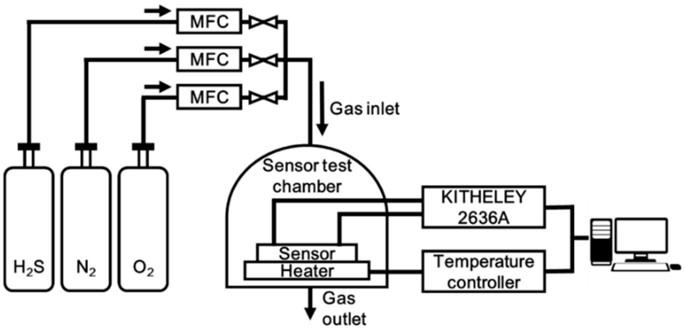

Figure S1. Schematic of gas sensing system.

H_2S concentration [ppm]	H_2S (N_2 balance, 100 ppm) [sccm]	O_2 (99.999%) [sccm]	N_2 (99.9999%) [sccm]	Total gas flow [sccm]
0.0 (dry air)	0.0	42.0	158.0	200.0
0.15	0.3	42.0	157.7	200.0
0.5	1.0	42.0	157.0	200.0
1.5	3.0	42.0	155.0	200.0
5.0	10.0	42.0	148.0	200.0

Table S1. Composition of gas mixture for various H_2S concentration.

Heat treatment temperature [°C]	Response [Rg/Ra]	Response time [sec]	Recovery time [sec]
400	1.34	512	1395
500	1.36	649	1090
600	2.36	99	536
700	3.62	169	432

Table S2. Characteristics of (**a**) CuO-05 sensors heat treated at different temperatures.

Sample ID	Response [Rg/Ra]	Response time [sec]	Recovery time [sec]
**CuO-005**	**1.26**	**351**	**1257**
**CuO-02**	**2.09**	**182**	**775**
**CuO-05**	**2.36**	**99**	**536**
**CuO-10**	**2.70**	**308**	**514**

Table S3. Characteristics of CuO sensors deposited at various deposition pressures and then heat treated at 600 °C.

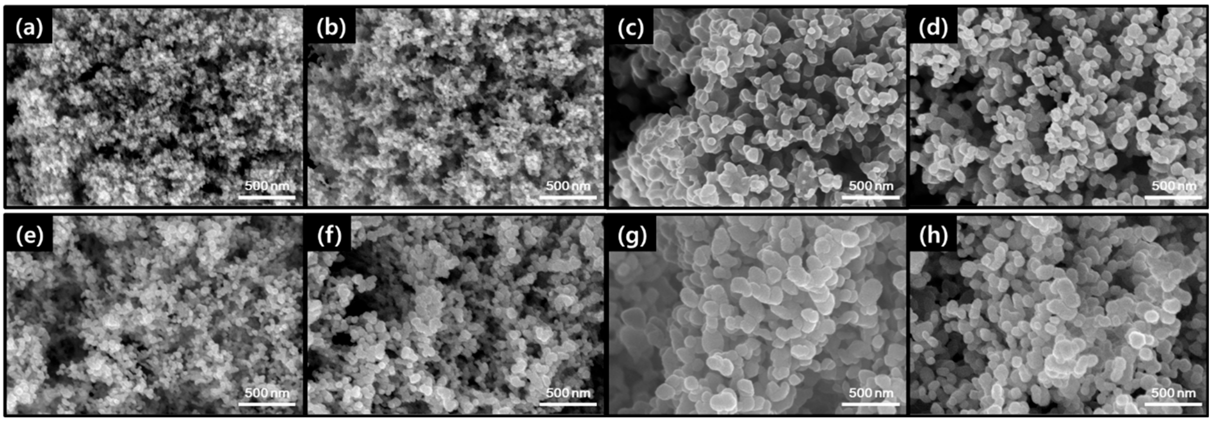

Figure S2. FE-SEM (**a**–**d**) surface and (**e**–**h**) cross-sectional images of CuO-05 films heat treated at 400, 500, 600, and 700 °C, respectively.

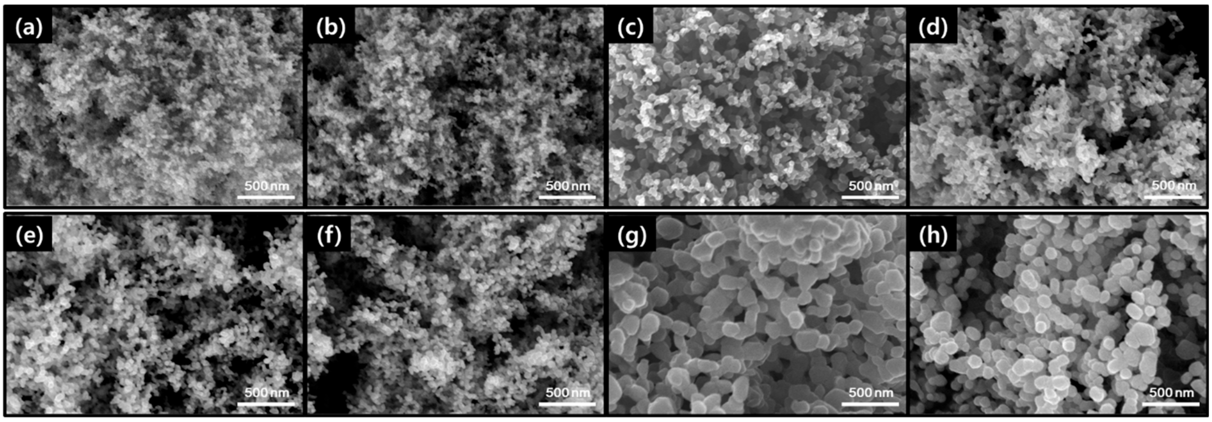

Figure S3. FE-SEM (**a**–**d**) surface and (**e**–**h**) cross-sectional images of CuO-10 films heat treated at 400, 500, 600, and 700 °C, respectively.

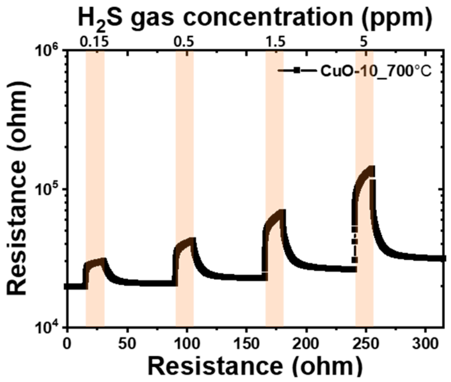

Figure S4. Resistance change graph for various H_2S concentrations of CuO-10 heat treated at 700°C (operating temperature: 325 °C).

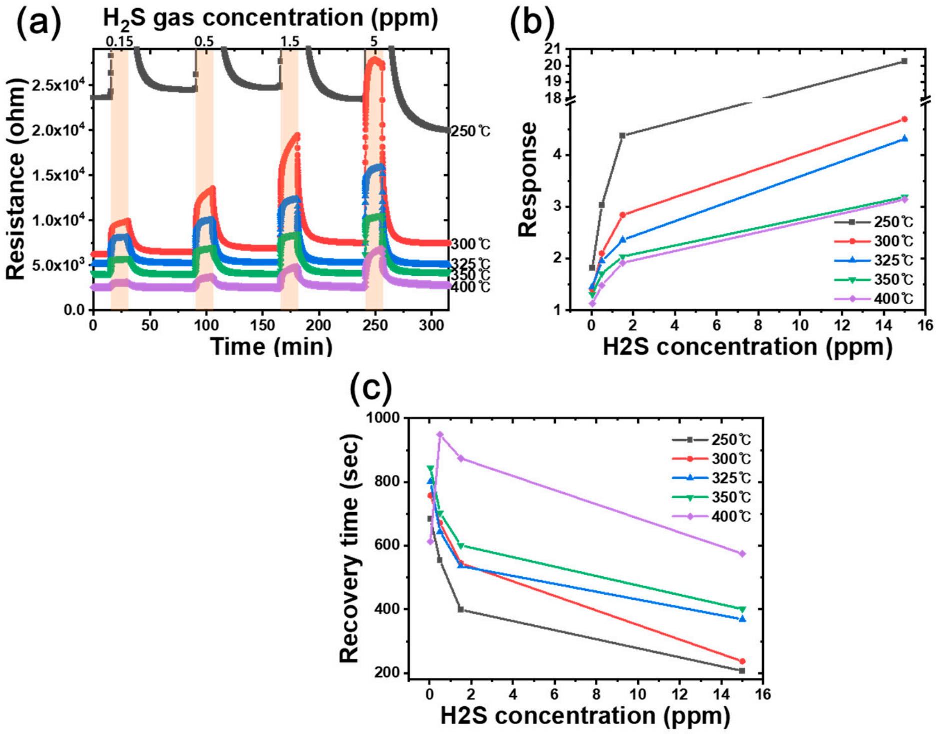

Figure S5. Sensor performance as a function of operating temperature of CuO-05 heat treated at 600 °C: (**a**) behavior, (**b**) response, and (**c**) recovery time for various H_2S gas concentrations.
